# Editorial: Network analysis of social media texts

**DOI:** 10.3389/frma.2025.1558798

**Published:** 2025-03-06

**Authors:** Ke Jiang, Weiai (Wayne) Xu, James Danowski

**Affiliations:** ^1^School of Communications, Elon University, Elon, NC, United States; ^2^Department of Communication, University of Massachusetts Amherst, Amherst, MA, United States; ^3^Department of Communication, University of Illinois Chicago, Chicago, IL, United States

**Keywords:** semantic network analysis, coherency network analysis, network analysis, social media text analysis, computational methods

Social media platforms have transformed communication by providing spaces for public discourse, activism, and knowledge-sharing. The vast amounts of unstructured text generated on these platforms reflect diverse perspectives, ideologies, and subjective opinions. Analyzing this data requires advanced methodologies capable of uncovering its complexity. The Research Topic “*Network analysis of social media texts*” explores how semantic network analysis can illuminate the structures and dynamics of social media discourse, offering valuable insights into this rapidly evolving field.

Over the past five decades, semantic network analysis has become a powerful tool for visualizing relationships, identifying patterns, and modeling communication networks. Unlike traditional data analysis, which often relies on structured formats, semantic network analysis excels at processing unstructured data, such as social media texts. This ability to analyze complex, real-time conversations makes it an ideal approach for understanding the dynamics of digital communication. The contributions in this Research Topic engage with issues like misinformation, polarization, crisis management, and social movements, advancing theoretical frameworks while offering practical insights for social media research.

The scope of this Research Topic is intentionally broad, addressing critical challenges faced by social media platforms today. By combining semantic network analysis with complementary methodologies, the studies offer fresh perspectives on these challenges. Together, they provide pathways for future research and suggest strategies for policymakers seeking to mitigate negative outcomes while amplifying the positive potential of these platforms.

Barnett et al. set the stage with a comparative analysis of topic modeling, community detection, and cluster analysis in extracting meaning from health-related social media datasets. Their findings underscore the clarity and interpretability of semantic network approaches, offering a methodological benchmark for analyzing complex datasets. This foundational work provides a framework for navigating the challenges of unstructured text data.

Jiang and Xu extend the conversation into digital activism, exploring the interplay between the hashtags #BlackLivesMatter and #StopAsianHate. Using coherency network analysis, they reveal the evolution and intersectionality of these movements, emphasizing shared goals of racial justice and solidarity. This study bridges theoretical insights with actionable strategies for understanding and amplifying online social movements.

Danowski et al. introduce Cascaded Semantic Fractionation (CSF), an iterative method for refining semantic domains in large-scale text analysis. By applying CSF to COVID-19-related discourse, the authors demonstrate its capacity to isolate relevant content and trace narrative shifts over time. This methodological innovation addresses limitations in traditional text analysis and equips researchers with a robust tool for exploring dynamic datasets.

Randazzo et al. focus on crisis communication, employing multilayer semantic network analysis to examine community tensions following Hurricane Ida. Their research delves into interconnected issues such as environmental sustainability, housing equity, and economic resilience. By highlighting grassroots narratives often overshadowed by institutional perspectives, their study offers fresh insights into how communities articulate challenges and responses during crises, providing practical implications for disaster communication.

Noakes et al. investigate the dissemination of a popular science article on Twitter, blending qualitative and semantic network analysis to examine how professional identities and localized content-sharing strategies shape communication. This interdisciplinary approach underscores the importance of contextual understanding in analyzing social media interactions, particularly in the context of science communication. Their findings highlight the value of combining computational and qualitative methods for richer, more actionable insights.

Collectively, these contributions illustrate the versatility and power of semantic network analysis in addressing multifaceted challenges associated with social media research. By uncovering hidden patterns, visualizing relationships, and exploring the dynamics of online interactions, these studies provide actionable insights for researchers, policymakers, and practitioners. For instance, Barnett et al.'s methodological framework helps researchers make informed decisions, while Danowski et al.'s CSF approach scales efficiently to large datasets. Similarly, Jiang and Xu's work illuminates the strategies behind digital activism, offering practical applications for policymakers and advocates.

Beyond academic contributions, these studies address real-world challenges. Randazzo et al.'s exploration of crisis communication offers guidance for community-centered approaches to disaster management. Noakes et al.'s findings on professional identities in science communication inform strategies for disseminating accurate, accessible information online. Together, these studies demonstrate how semantic network analysis can help address misinformation, polarization, and crisis management while fostering constructive dialogue and collaboration.

Looking ahead, this Research Topic highlights promising directions for future research. One avenue is integrating machine learning with semantic network analysis to automate and scale pattern detection in large datasets. Cross-platform analyses also hold potential, offering insights into how narratives and interactions evolve across different social media ecosystems. These approaches can uncover broader trends and connections that may be missed in single-platform studies.

The combination of computational and qualitative methods, as demonstrated by Noakes et al., represents another critical direction. Bridging these approaches allows researchers to incorporate both data-driven insights and contextual nuances, enriching analyses and enhancing the applicability of findings. Emerging areas such as influencer behavior, collective intelligence, and cross-cultural comparisons also provide fertile ground for advancing the field of social media research.

The Research Topic “*Network analysis of social media texts*” underscores the transformative potential of semantic network analysis in unraveling the complexities of social media communication. By addressing critical questions and employing diverse methodologies, this Research Topic provides a roadmap for future research that continues to innovate and expand the boundaries of this dynamic field. Ultimately, it contributes to the broader goal of fostering informed, inclusive, and collaborative digital communities, harnessing the power of semantic network analysis to drive meaningful change in a digitally connected world.

